# Glass eels (*Anguilla anguilla*) imprint the magnetic direction of tidal currents from their juvenile estuaries

**DOI:** 10.1038/s42003-019-0619-8

**Published:** 2019-10-08

**Authors:** Alessandro Cresci, Caroline M. Durif, Claire B. Paris, Steven D. Shema, Anne Berit Skiftesvik, Howard I. Browman

**Affiliations:** 1 Department of Ocean Sciences, Rosenstiel School of Marine & Atmospheric Science, 4600 Rickenbacker, Causeway, FL 33149-1098 USA; 2 Institute of Marine Research, Austevoll Research Station, Sauganeset 16, N-5392 Storebø, Norway; 3 Grótti ehf., Grundarstíg 4, 101 Reykjavík, Iceland

**Keywords:** Animal migration, Behavioural ecology

## Abstract

The European eel (*Anguilla anguilla*) hatches in the Sargasso Sea and migrates to European and North African freshwater. As glass eels, they reach estuaries where they become pigmented. Glass eels use a tidal phase-dependent magnetic compass for orientation, but whether their magnetic direction is innate or imprinted during migration is unknown. We tested the hypothesis that glass eels imprint their tidal-dependent magnetic compass direction at the estuaries where they recruit. We collected 222 glass eels from estuaries flowing in different cardinal directions in Austevoll, Norway. We observed the orientation of the glass eels in a magnetic laboratory where the magnetic North was rotated. Glass eels oriented towards the magnetic direction of the prevailing tidal current occurring at their recruitment estuary. Glass eels use their magnetic compass to memorize the magnetic direction of tidal flows. This mechanism could help them to maintain their position in an estuary and to migrate upstream.

## Abstract (web summary)

Cresci et al. show that glass eels are able to use their magnetic compass to imprint the magnetic direction of tidal flows at the estuaries of the streams where they develop into yellow eels. The authors hypothesize that this ability helps glass eels to maintain their position in an estuary and to migrate upstream.

## Introduction

**Fig. 1:**
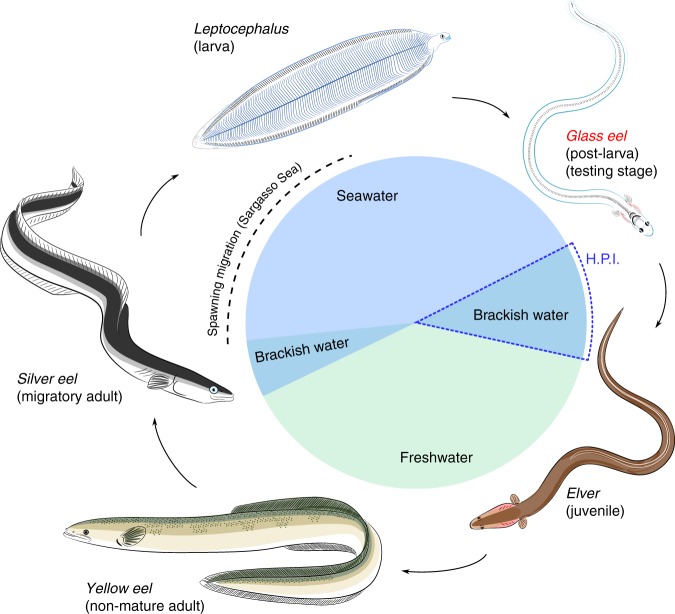
Life history of the European eel (*Anguilla anguilla*). Eels hatch as leptocephalus larvae in the Sargasso Sea. As larvae, they drift across the Atlantic Ocean to the continental slope of Europe, where they metamorphose into post-larval, transparent glass eels. The glass eels migrate across the continental shelf and eventually reach the brackish water of estuaries. After metamorphosing into pigmented juveniles, called elvers, they start the ascent into freshwater, where they will grow into adult yellow eels. After some years, yellow eels undergo another metamorphosis into silver eels, which migrate for thousands of kilometers to the Sargasso Sea where they spawn and die. Eels used in this study were at the stage of glass eel (red font), and the Hypothetical Period of Imprinting (H.P.I.) is highlighted by a dashed blue polygon. Artwork credit A. Cresci

The European eel (*Anguilla anguilla*) is a migratory species that crosses the Atlantic Ocean twice during its life (Fig. 1). After hatching in the Sargasso Sea^1^, eel leptocephali larvae move with the gulf stream more than 5000 km until they reach the continental slope of Europe^2,3^. There, they metamorphose into the post-larval transparent glass eel^4^. At this stage, glass eels migrate across the continental shelf to the coast^2,5^. After reaching the coast, glass eels enter estuaries, where some of them continue their migration upstream into freshwater^6^. The eels that enter freshwater spend most of their lifetime (5–25 years) there, growing first into the adult yellow eel stage, and then into silver eels^2^. Silver eels then navigate back to the Sargasso Sea where they spawn and die^1,7,8^.

The European eel is a commercially important species that is critically endangered [International Union for Conservation of Nature (IUCN)]: eel populations have declined precipitously since the 1980s^9–11^. Much research on the conservation and management of eels has been undertaken^12,13^, driven to some extent by the requirement for member states of the European Union to develop management plans for the recovery of eel populations. The arrival of the early-life stages to estuaries (recruitment), is an important phase of their migration, and the number of recruiting glass eels have been consistently declining over the past decades^10,14^. Thus, a better understanding of the dynamics of this step of the migration, including deeper knowledge of the orientation mechanisms involved, is needed.

Glass eels use multiple spatial and sensory cues for orientation^15–17^, which could hypothetically be imprinted and used years later during their return journey to the spawning areas. It is possible that eels imprint spatial information from the environment at several steps during their migration, such as at the time of hatching or during the journey to the European coast. Imprinting is associated with a broad variety of processes in animals, including food and habitat preference, host selection in parasitic animals, and homing^18–20^. Imprinting is a fast-learning process that typically occurs during a specific life history phase^18,19,21^. Imprinting of spatial cues, such as olfactory cues in salmon^22^, occurs in several aquatic animals. Animals can also imprint information from the magnetic field of the Earth. Aquatic species such as pacific salmon (*Oncorhynchus nerka*)^23^ and sea turtles (*Caretta caretta*)^20^ imprint specific features of the Earth’s magnetic field and use this to orient during migration. The European eel is a long distance migrator that also uses magnetic fields to orient during migration^24,25^. However, whether imprinting of magnetic cues occurs, either at the larval or glass eel stage (or both), remains unknown.

Glass eels recruit at tidal estuaries along the European coast, where they are exposed to the alternation of ebbing and flooding tidal currents. In aquatic environments, fish display an unconditioned response to water currents (rheotaxis), which can be positive (the fish swims into the current) or negative (the fish swims in the same direction as the current)^26,27^. Rheotaxis is a major component of fish orientation in tidal estuaries in which currents can be fast and visibility low^27^. In such situations, fish use rheotaxis for both upstream migration and upstream-oriented station holding behavior to minimize energy expenditure in flowing water^28^. Glass eels display both of these rheotactic behaviors when they recruit to estuaries, showing rhythmic patterns of positive or negative rheotaxis synchronized to the tidal phase^29^. This tidal-dependent orientation is important during migration, as glass eels exploit tidal flows to enter brackish and freshwater using selective tidal stream transport (STST)^30–33^. This constitutes a deeply rooted behavior in many species of fish and invertebrates inhabiting tidal estuaries^34^.

In order to orient with respect to water flow, fish rely on multiple sensory cues, including visual, vestibular, and tactile^35,36^. Rheotaxis in fish is also influenced by changes in the magnetic field at intensities as low as the Earth’s^37,38^, suggesting that magnetic fields could be one of the reference cues that fish use to orient in the presence of flowing water. Glass eels exhibit rheotaxis in the presence of tidal currents and sense magnetic cues: our previous work showed that glass eels use the magnetic field for orientation using a magnetic compass mechanism, switching magnetic direction according to an endogenous tidal rhythm in the absence of a current^16^. Specifically, eels oriented to the magnetic south with the ebb tide and to the north with the flood tide^16^. While these results show that glass eels use a magnetic field-based compass mechanism, the stimuli that cause glass eels to swim toward specific magnetic directions at this stage of the migration are unknown.

We tested the hypothesis that the magnetic orientation direction of glass eels (i.e., the orientation direction relative to the direction of the magnetic field) is imprinted from the tidal currents at the estuaries where they recruit. To test this hypothesis, 222 glass eels were collected at stream estuaries flowing toward different cardinal directions (north, southeast, south, and northwest). The magnetic orientation of glass eels was observed in a magnetic laboratory facility, using an electric coil system that modified the direction of the magnetic field, while depriving the glass eels of any other external cues that they could use for orientation. We show that glass eels orient toward the magnetic direction of the prevailing tidal current occurring at their estuary. This is interpreted as evidence that glass eels form and retain a magnetic memory of tidal flows.

## Results

### Tests in the magnetic laboratory

The magnetic orientation behavior of glass eels was observed following the methods described in our previous work (see the Methods section)^16^. In brief, a circular transparent chamber (Drifting In Situ Chamber, DISC; Supplementary Fig. 1b)^39^ was submerged in a circular black tank in a magnetic laboratory (MagLab; https://fishlarvae.org/facilities/magnetoreception-test-facility/) located in Austevoll, Norway (60.1175 N, 5.2118 E; Supplementary Fig. 1). The MagLab is equipped with a triaxial electric coil system that allowed us to manipulate the magnetic field to which glass eels were exposed in the experimental tank.

**Fig. 2:**
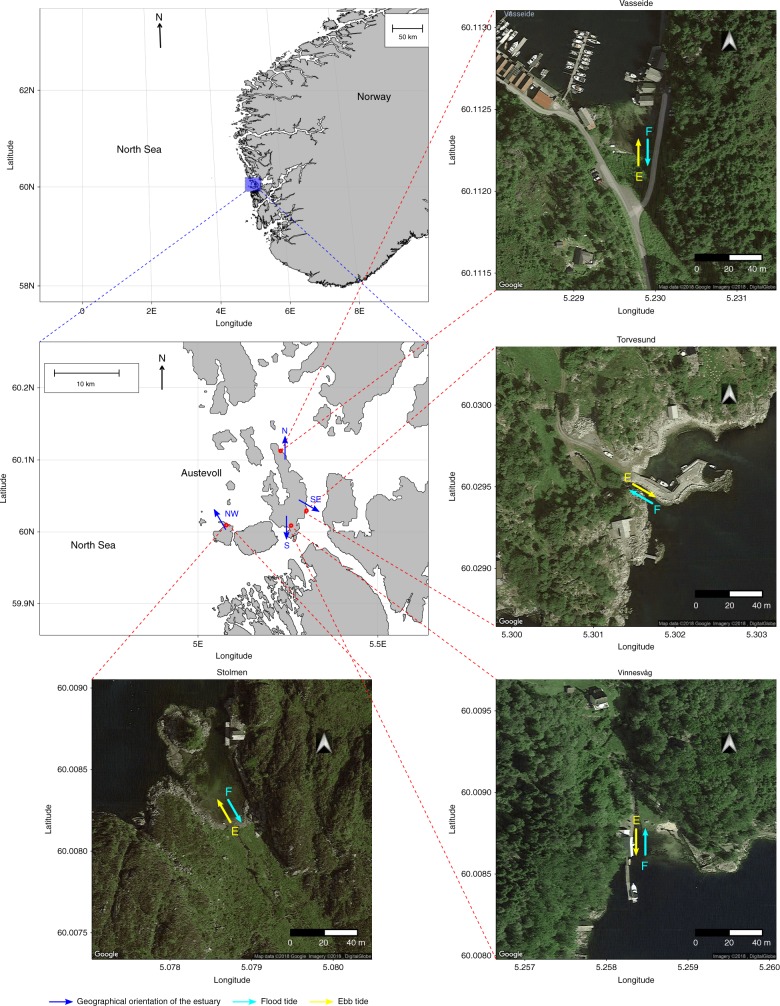
Estuaries where glass eels (*Anguilla anguilla*) were collected and the direction of tidal currents. Maps show Norway (upper left) and the Austevoll archipelago. Red points show the location of the estuaries. Blue arrows and blue cardinal points show the magnetic direction toward which the estuaries flow; arrows start from the freshwater side, and point toward the seawater side. The satellite images show the aerial view of each one of the four estuaries. Sky-blue and yellow arrows show the magnetic direction of the tidal currents at each of the estuaries (E = ebb current, F = flood current)

Glass eels were collected at four stream estuaries located around the Austevoll archipelago (Fig. 2), before they migrated into freshwater. The tidal estuaries were selected according to their geographical features, as we searched for estuaries flowing toward different cardinal directions. Glass eels were collected at Vasseide (60.1122 N and 5.2298 E, flowing to the north), Torvesund (60.0294 N, 5.3016 E, flowing to the southeast), Vinnesvåg (60.0088 N, 5.2583 E, flowing to the south), and Stolmen (60.0082 N and 5.0788 E, flowing to the northwest) (Fig. 2). Upstream of these tidal areas (for example, in Torvesund, where the stream changes direction), there was no action of the tides and only pigmented elvers were present. The data set used in this study is composed of the orientation data of 222 glass eels. These include the re-analysis of the orientation data from our previous work of 49 glass eels tested in the MagLab in 2015 (eels coming from Vasseide)^16^, and 173 glass eels newly collected from Vinnesvåg, Torvesund, and Stolmen (S, SE, and NW oriented estuaries; Table 1).

**Table 1:** The estuaries where the glass eels (*A. anguilla*) *were* collected are listed

Estuary	Orientation of the estuary	*n*	Dates of the tests
*Vasseide*	N	49	16–22 April 2015
*Vinnesvåg*	S	36	9–16 May 2016
*Torvesund*	SE	24	13–16 May 2016
*Stolmen*	NW	113	28 April–18 June 2017

Orientation of the estuary is the cardinal direction toward which each of the estuaries flows. *n* number of glass eels tested in the magnetic laboratory listed by stream of provenience. The dates of the tests are also indicated

Glass eels were individually exposed to different configurations of the magnetic field: the magnetic north in the laboratory was rotated toward one of the four cardinal points of the Earth’s magnetic field, and each eel was exposed to only one of the four magnetic conditions used in this study (Supplementary Fig.  2). The orientation of each glass eel was then assessed with respect to the rotated magnetic north in the laboratory (Supplementary Fig. 2).

Each eel was tested in the magnetic lab between the peaks of high and low tide, during one of the rising/lowering tides (ebb/flood) occurring along the Austevoll archipelago. The magnetic orientation direction of each eel was then analyzed with respect to the magnetic direction of the tidal current occurring contemporaneously at the eel’s recruitment estuary. Thus, in our reference system, we considered the magnetic direction of the tidal flow with respect to the Earth’s magnetic field) as 0°. Finally, we computed the angular difference between the magnetic orientation direction of each eel and the magnetic direction of the tidal flow.

### Magnetic orientation

**Fig. 3:**
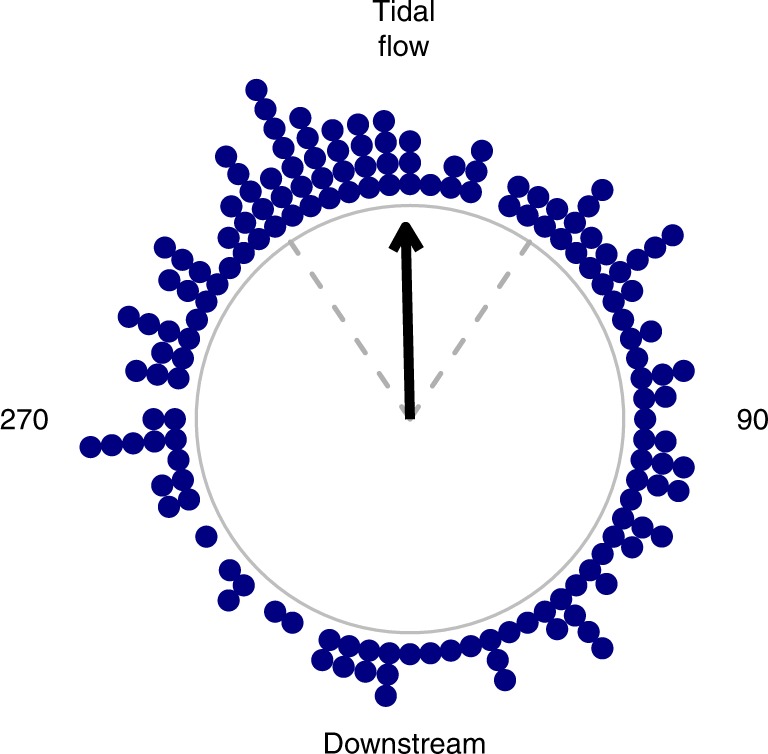
Magnetic orientation of glass eels (*Anguilla anguilla*) with respect to the magnetic direction of the tidal flows. In the circular plot, the outer gray circle represents the *x-*axis. The angle between the magnetic orientation of each glass eel that significantly oriented (Rayleigh’s *p* < 0.05 on individual tracks) and the direction of the tidal flow (top of the plot) is shown as a navy-blue data point (N = 155). The bottom of the plot represents the downstream direction of the flow. Significant (Rayleigh’s *p* < 0.05) collective orientation toward the upstream magnetic direction of the tidal flow is shown as a black arrow pointing toward the top of the plot. The direction of the arrow point toward the mean orientation direction of the glass eels. Dashed gray lines show the 95% confidence interval around the mean direction of the eels. For clarity, the data are displayed binned by 5°

The overall proportion of glass eels showing a preferred magnetic orientation direction was 70% (155 out of 222). This proportion, however, changed depending on the estuary, but it was always >50% (Table 2). Glass eels significantly oriented to the magnetic direction of the prevailing tidal flow that was occurring at their recruitment estuaries during the tests. Their average magnetic orientation direction was 359° (N = 155, Rayleigh’s *p* = 0.0018, *r* = 0.2; Fig. 3), and it matched the magnetic direction of the incoming tidal flow (0°, Fig. 3).

**Table 2:** Proportion of glass eels (*A. anguilla*) showing a significant magnetic orientation direction

Estuary	*n*	Orienting glass eels	Proportion of orienting eels
*Vasseide*	49	35	71%
*Vinnesvåg*	36	20	56%
*Torvesund*	24	15	62%
*Stolmen*	113	85	75%
Total	222	155	70%

*n*: the number of glass eels tested in the magnetic laboratory listed by stream of provenance. The Table shows the number of glass eels displaying significant orientation and the proportion of eels that oriented

## Discussion

Glass eels use their magnetic compass to memorize the magnetic direction of the currents at the estuaries where they recruit. This is evidence that these fish are capable of forming and retaining a magnetic memory of the direction of water currents, and to use it to orient in moving water during migration.

Satellite tracks of loggerhead sea turtles (*Caretta caretta*) and fur seals (*Callorhinus ursinus*) indicate that they detect the downstream direction of currents in open waters, implying the use of magnetic cues^40,41^. It was also hypothesized that elasmobranchs, that navigate by detecting electromagnetic fields^42^, could sense the direction of oceanic currents through the electricity produced by the friction of water moving over the sea bottom^43^. When visual reference points were present, the interaction between the magnetic sense and orientation to water flows was observed in shoaling zebrafish (*Danio rerio*), which change their orientation in flowing water according to the direction of the magnetic field^37^.

The results described in this study add evidence that eels detect and form a memory of the magnetic direction of currents, supporting the possibility that these fish—and possibly others—have the sensory capacity to integrate magnetic and rheotactic information and use them for orientation. Analogous results involving learning of magnetic cues were reported at a later stage in the life cycle of the eel: silver eels displaced between different locations learned the compass direction of their displacement^24^. In that study, orientation was dependent on temperature, demonstrating their ability to modulate their response to the magnetic field according to other environmental signals.

The magnetic memory of the flows at the recruitment estuaries described in this study might represent a specific case of imprinting. In its original definition^21^, imprinting describes a learning process that occurs in a restricted, sensitive time of the life of an animal, and that this memory is stable and retained over time. Considering the glass eels, it is possible that they imprint the rhythm of the tide and the magnetic direction of flows during a specific, possibly sensitive period, when they first enter the estuary, which constitutes a life history transition from seawater to freshwater physiology. Previous studies on salmon smolts show that imprinting can occur in such transition periods associated with surges in plasma thyroxine^44^. Furthermore, otolith analyses revealed that the time that glass eels spend at the estuaries could be very long as some never enter freshwater^6^. Thus, this memory could be retained and used for a significant portion of the life of a glass eel, before the elver stage. However, it is also possible that these results may represent a phenomenon of continuous ongoing learning, and that this magnetic memory of flows could remain flexible over time. For example, eels might adjust their magnetic heading to varying hydrodynamic conditions at the estuary, or further upstream, where the magnetic direction of the current could change. These are interesting scenarios that we intend to address in future work. Furthermore, the estuaries selected in this study are quite rectilinear systems, but it is possible that highly curvilinear estuaries are harder for glass eels to navigate and future work should investigate whether this affects recruitment to such sites.

In movement ecology, the concept of imprinting has been mostly associated with the return of animals to natal areas (homing). Among aquatic species, the most studied case is that of salmon. As smolts, salmon record the specific combination of odors from their natal stream, which they will use as adults to guide their spawning migration upstream^22,45^. In addition to olfactory cues, there is evidence that salmon imprint magnetic information when they make first contact with seawater, which they use later in life to find the coastal area where their natal estuary is located^23^. Similarly, sea turtles imprint the geomagnetic field associated with their natal beach, which they use years later to return and nest^20^. In the case of the eel, the magnetic imprinting observed in glass eels has a different importance for their migration. Glass eels learned the magnetic direction of currents at their recruitment estuary. This estuary will be the same as that which they encounter years later during the descent from the stream to start their oceanic spawning migration as adult silver eels. Whether eels keep a magnetic memory of their estuary through adulthood, and whether this constitutes a reference to start the marine phase of the spawning migration in the right direction, is unknown. For example, it is possible that adult eels recognize the direction of the alternating tidal flows experienced as glass eels when they descend the estuary, and that this constitutes a trigger to undertake the last physiological and behavioral changes at the start of their long marine migration. However, this hypothesis needs to be tested in future work.

The use of the magnetic sense to imprint the direction of currents at estuaries could also help glass eels at this earlier phase of their migration. During their estuarine residency, glass eels undergo physiological and morphological changes, developing pigmentation, developing jaws and teeth, and adapting to freshwater^2^. Thus, glass eels need to maximize the energetic efficiency of their swimming behavior at the estuary, both to keep their position and to migrate upstream^46^. In environments such as streams, rivers, or estuaries, fish orient with respect to the current by using visual and tactile cues (such as the bottom)^35,47^. Our results show that glass eels also use their magnetic sense as a reference to orient against the direction of the flow. This could help them in several ways, such as keeping the right compass course when visual reference points are lost or obscured (turbid water in rivers or muddy streams), or when there is no physical contact with the substrate. This would be of great importance especially for glass eels recruiting to large, long tidal estuaries such as the Gironde in France, where the effect of the tidal flows extends for kilometers. Moreover, in such large estuaries the water is turbid, and temperature and salinity are subjected to high variability. Thus, the use of the magnetic compass system described in this study would have obvious advantages in such environments, providing a fixed reference for orientation.

Although glass eels oriented along the magnetic direction of tidal currents at their recruitment estuaries, there was unaccounted for interindividual variability (Rayleigh’s *r* = 0.2). Variation in orientation response is dependent on the internal state (or behavioral traits) of the animals, which significantly contribute to their decision to move^48^. The internal state (often classified as proactive and reactive, or migratory and nonmigratory phenotypes) plays an important role in the magnetic sense of fish, up to the point that it can make the difference between responding or not responding to magnetic stimuli^38^. European eels display significant differences among individuals concerning their tendency to migrate. Experiments using flume tanks showed that glass eels (*A. anguilla*), elvers, and yellow eels display different tendencies to migrate and can be separated into migrants that actively move upstream or downstream and nonmigrant that do not show a particular tendency to migrate^46,49^. It is possible that those glass eels not showing any preferred magnetic orientation direction have a nonmigrant phenotype. Future work should repeat these experiments on magnetic imprinting after dividing the glass eels according to their motivation to migrate and dividing migrating glass eels into upstream vs. downstream migrants. This could be accomplished using an experimental setup similar to that used by Bureau Du Colombier et al., with a flume tank connected to upstream and downstream traps^46^.

Glass eels learn the features of the environments that they encounter during migration. Future work should investigate whether migrating silver eels that are translocated at the glass eel stage have more difficulty in finding their way toward the sea compared with nontranslocated ones. If they do, then the results of this study would be relevant to management plans that include the restocking of glass eels in freshwater where the population is most depleted^46^.

## Methods

### Animals and maintenance

The glass eels were collected in Norway, before their upstream freshwater migration between March and June of 2015, 2016, and 2017. They were caught using hand nets searching under rocks and sediment at low tide. None of the animals used in this study were pigmented (developmental stage: V–VI 2), and they did not have food in the gut. Glass eels were kept in 20 -L maintenance tanks, where they were re-acclimated to near full salinity seawater (32 ppt) after capture. They were kept in aerated aquaria in a temperature-controlled room set to ambient conditions similar to those of the local Langenuen fjord (ranging between 6 and 10 °C). Animals were not fed (they were at the pre-feeding stage) and were kept in 14 h light and 10 h dark cycle (following the daylength at the study location during the observation period). Two-thirds of the volume of each aquarium were replaced with filtered seawater every 48 h to maintain water quality. The seawater was provided by the filtration system at the Institute of Marine Research’s Austevoll Research Station, which collects seawater from the Langenuen fjord at a depth of 160 m. Before the tests, glass eels were taken from the large aquaria and placed in individual 500 -mL white plastic containers filled with seawater at the same temperature as the aquaria and transported to the magnetic laboratory (MagLab) in a cooler to keep the temperature stable. No permits were required by the Norwegian authorities because no glass eels (*Anguilla anguilla*) were harmed while performing the experiments, and after use, they were either returned to the wild or killed using an approved method. Eels were collected under research catch permit #11/11448 issued by the Norwegian Fiskeridirektorat.

### Experiments and data analysis

The experiments in the laboratory followed the same protocol as described by Cresci et al.^16^. The MagLab is designed to study the magnetic orientation of aquatic animals. It is equipped with a triaxial electric coil system (Supplementary Fig. 1c), with a design described by Merritt et al.^50^. The coils are connected to a power supply (max. 3 A). A black circular fiberglass tank (diameter, 1.40 m; height, 0.90 m; see Supplementary Fig. 1c) filled with seawater is located at the center of the coils. The seawater is pumped from the sea (300 m away). The building (see Supplementary Fig. 1a) is made of nonmagnetic material, and is distant from any magnetic interference (163 m from the nearest electrical disturbance and 365 m from the closest building; Supplementary Fig. 1a).

For this study, we used the DISC (“Drifting In Situ Chamber”, Supplementary Fig. 1b)^39^ as a behavioral chamber, submerged in the circular dark tank (see Supplementary Fig. 1c). The chamber of the DISC in which individual glass eels swam one at a time was 40 -cm wide (diameter) and 15 cm deep. The chamber was semi open, as the bottom was rigid and made of acrylic, while the walls were made of transparent fine mesh, which allows water and dissolved gas exchange. The top of the chamber was covered with opaque white plastic, which diffused light uniformly in the chamber. Light intensity in the tank was low (0 lum/ft^2^ from HOBO light sensor on the bottom plate of the DISC frame).

The behavior of glass eels in the DISC was observed using a GOPRO HERO 4 camera placed on the bottom plate of the DISC acrylic frame, underneath the chamber (Supplementary Fig. 1b, d). The DISC was equipped with an analog compass attached to the acrylic poles of the DISC frame and placed below the circular arena. This positioning eliminates the possibility that the compass would be a visual reference for the eel.

The MagLab has two nested electric coil systems. We used one of them to cancel out the horizontal component of the ambient field. We used the second system to create a magnetic field of 48.8 to 50 µT, which is the same total intensity as the ambient field, and to rotate the magnetic north. We did not change the intensity and inclination inside the coil compared with the ambient field (48.8 to 50 µT and 73°, with a deviation of <1°).

We recorded each eel for 15 min, with the first 5 min as an acclimation period^16,51^. We simulated four magnetic field conditions, with the magnetic north rotated towards the east, south, west, or north of the geomagnetic field (see Supplementary Fig. 2 and Supplementary Data 1). We exposed each glass eel to only one of the four conditions. This technique eliminated any nonmagnetic bias that could have influenced the orientation response of the animals. The MagLab is a bespoke facility that was designed to study magnetic orientation in marine organisms and to eliminate other environmental cues (water flows, odor plumes, sunlight, or any celestial cues). The walls are constructed of aluminum and wood, and they are insulated to protect from sound and temperature fluctuation. Every metal fitting, screw and bolt, is made of nonmagnetic material (aluminum, brass, or high-quality stainless-steel). The experimental room is completely isolated, and the top of the facility has a roof (i.e., no celestial cues). The facility is located at the top of a fjord, hundreds of meters from electromagnetic disturbances. To water flow in the tank, no pumps, aerator, or ventilation systems are present. The observation tank is supported on an autonomous concrete block, separating it from the rest of the building and isolating it from vibrations.

All tests were conducted during daytime. The data about the tide were obtained from the Norwegian Mapping Authority (www.kartverket.no). The direction of flow in the estuaries was assessed in situ using an analog compass.

The orientation of glass eels was determined through the analysis of the GOPRO images, tracking the position of the head of the eel in the circular arena every second for 10 min. Magnetic north had a different orientation in the laboratory during each test, and the position of the eels with respect to the magnetic north in the laboratory was monitored using the analog compasses. The video frames were processed using the DISCR tracking procedure, utilizing R and a graphical user interface provided by imageJ software^52,53^. Using this tracking procedure, we collected the positional data (in units of magnetic degrees) of the glass eel with respect to the center of the chamber, which were considered as bearings. The code utilized is available at the web page Drifting In Situ Chamber User Software in R (https://github.com/jiho/discr written by Jean-Olivier Irisson (Université Pierre et Marie Curie UPMC), released under the GNU General Public License v3.0.

We assessed the mean orientation of each individual from the bearings collected using the video tracking analysis (Supplementary Fig. 3). The mean of 600 data points, which represent the bearings of the eel in the chamber at each second (one position/s over 10 min period), was considered to be the orientation of each individual^51,52^. The orientation of each eel was then corrected with respect to the rotated magnetic North induced with the coils in the laboratory (Supplementary Fig. 3). As last step, the magnetic orientation was corrected with respect to the magnetic direction of the prevailing current occurring at the estuary where the eel was collected from (Supplementary Figs. 3, 4).

### Statistics and reproducibility

If the eel displayed a magnetic bearing in the chamber of the DISC, we considered it as indicative of directionality^39,52^. Whether the directionality was significant was assessed with the Rayleigh’s test of uniformity for circular data^52–54^. An outcome was considered statistically significant when *p* < 0.05 (alpha = 0.05)^52^. This analysis was performed on 222 glass eels tested individually.

After having assessed the orientation of each individual, the following step of the analysis evaluated whether the eels tested in the DISC show a significant pattern in their orientation, or whether they go toward a common direction. To assess collective patterns in orientation direction, we used the same statistical test, the Rayleigh test of uniformity on all the mean individual bearings of the eels that displayed orientation (N = 155), testing whether the frequency distribution of the directions displayed by the individuals was significantly different from random (95% confidence interval, alpha = 0.05)^52^. Possible effects on the results caused by the larger sample size of the eels collected in Stolmen was assessed statistically (analysis and results are displayed in Supplementary Fig. 5).

### Reporting summary

Further information on research design is available in the Nature Research Reporting Summary linked to this article.

## Supplementary information

Supplementary Information

Description of additional supplementary items

Supplementary data 1

Supplementary data 2

Reporting Summary

## Data Availability

All of the data displayed in Fig. 3 are available in the Supplementary Data 2.
