# Supplementary material for: Glass eels (Anguilla anguilla) imprint the magnetic direction of tidal currents from their juvenile estuaries
Source: Commun Biol. 2019 Oct 8;2:366. doi: 10.1038/s42003-019-0619-8 (PMC6783477; doi:10.1038/s42003-019-0619-8)
Supplement: Supplementary file 1 — Supplementary Information [file 42003_2019_619_MOESM1_ESM.pdf]

Supplementary Information

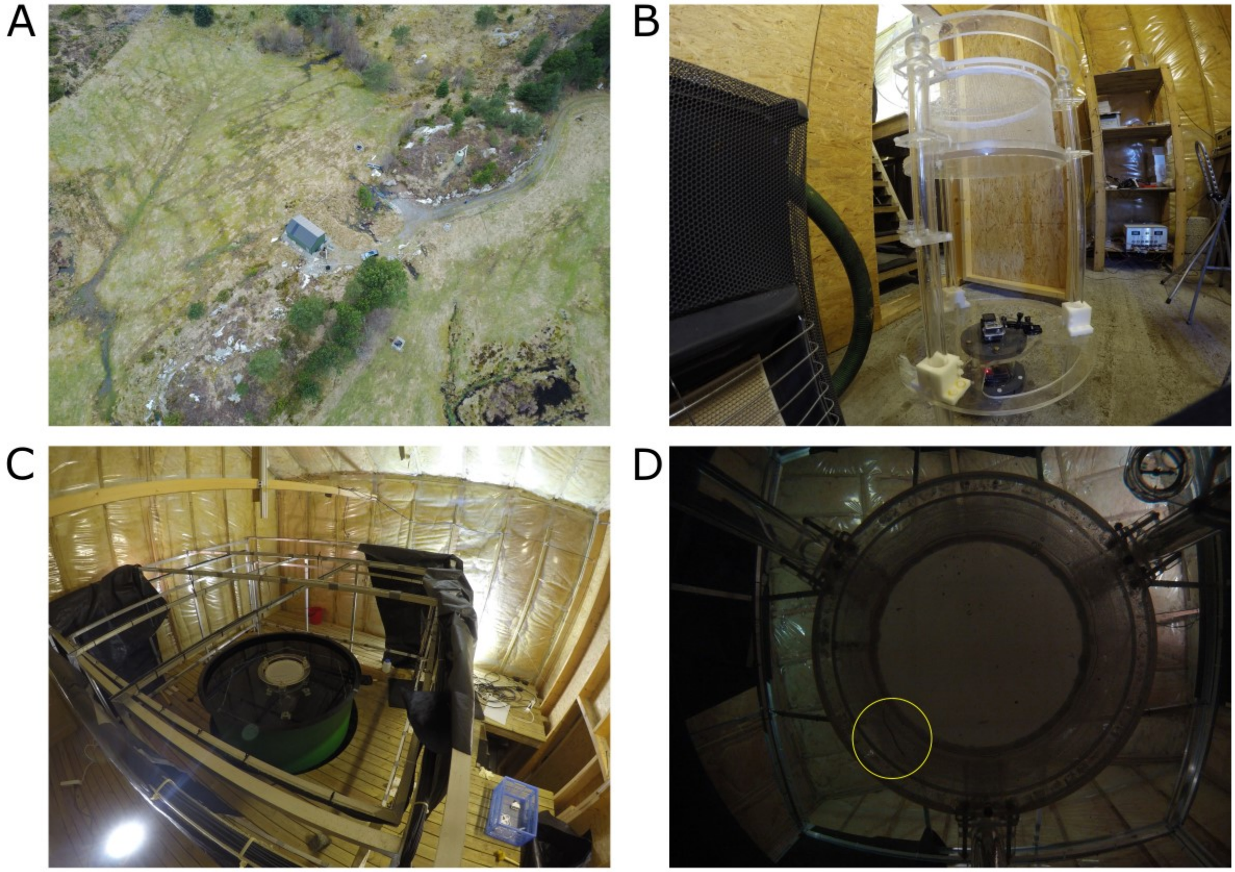

Supplementary Figure 1. Magnetic laboratory and DISC. A. Aerial view of the magnetic laboratory (MagLab). The aerial view makes clear that the MagLab is far from any electromagnetic disturbance. **B.** behavioral chamber (DISC) used in the study. During the tests, a white plastic board was placed on top of the chamber (visible in panel C). **C. Wire-wrapped** coils surrounding the experimental tank. The DISC was placed at the center of the tank. **D.** View from the GOPRO placed underneath the DISC chamber. The glass eel is highlighted by the yellow circle.

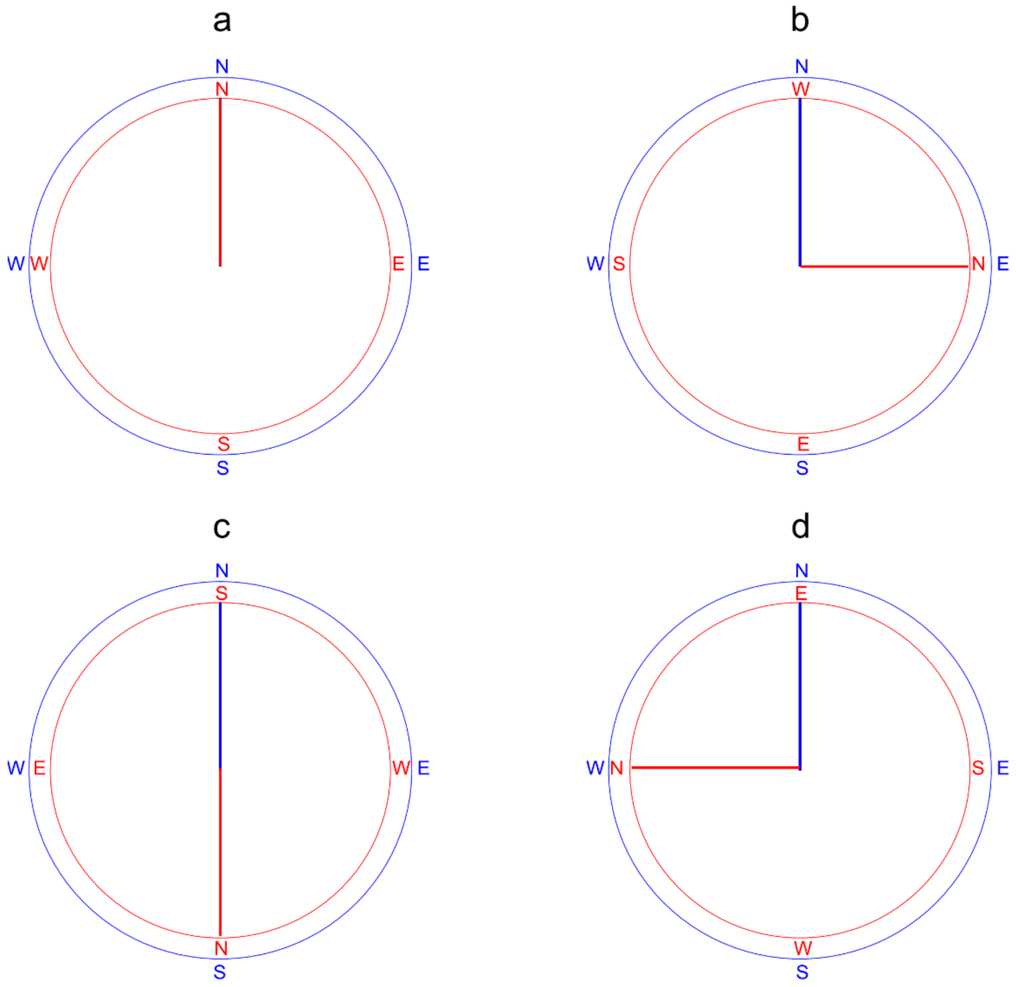

Supplementary Figure 2. Schematic diagram of the magnetic protocol. Blue N, E, S, W represent the Earth's magnetic cardinal points. Red N, E, S, W correspond to the magnetic cardinal points in the testing tank. The red line highlights the orientation of the magnetic North and the blue line the orientation of the Earth's North. Each eel was observed in one of these 4 magnetic conditions. The orientation was then assessed with respect the North in the tank (red North).

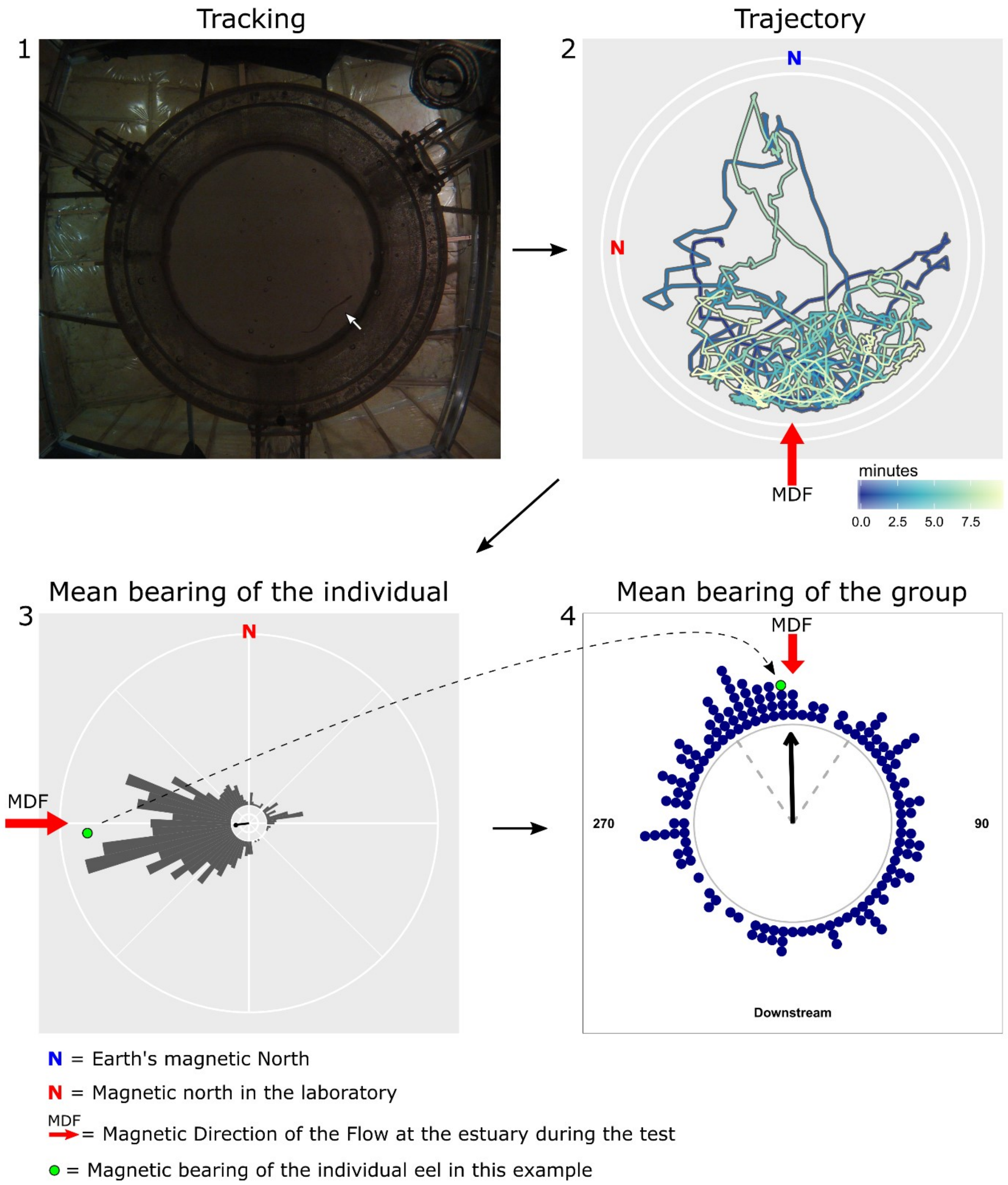

Supplementary Figure 3. Description of the analysis of the data collected with the DISC in the MagLab. **Step 1:** The first step is to collect data on the position of the glass eel (*Anguilla anguilla*) in the DISC using a tracking procedure conducted on the videos recorded during 1 test in the magnetic laboratory. The position of each glass eel is tracked every second for 10 minutes (i.e. 600 data points are collected per each glass eel tested). The photo shows an example of the view from underneath the chamber of a glass eel (highlighted by a white arrow) in the DISC. **Step 2:**

The trajectory of the glass eel is calculated from the datapoints collected through the video tracking. In this figure, the Earth's magnetic North, the rotated magnetic North in the laboratory and the Magnetic Direction of the tidal Flow occurring at the recruitment estuary (MDF) are shown. Notice that MDF is considered with respect to the North in the lab (red North), and in this example the flow at the estuary comes from the magnetic West. **Step 3:** The angle of each of the 600 data points with respect to the magnetic North in the lab and the center of the chamber is considered as a bearing. The mean orientation of the glass eel is assessed by applying Rayleigh's test of uniformity on the 600 bearings. If the outcome of the statistical test is significant ($P < 0.05$), the mean bearing (GREEN circle in this example) is considered as the preferred magnetic orientation direction of the glass eel. This mean orientation direction is then corrected with respect to MDF. **Step 4:** The last step of the analysis is performed on all the mean magnetic orientation directions of the glass eels tested after being corrected to MDF. At this step, the Rayleigh's test is applied on all the mean magnetic orientation directions. Through this step it is possible to assess whether glass eels had the tendency to orient towards a common direction. In this panel of the figure the GREEN circle corresponds to the mean magnetic orientation of the glass eel used as an example in the previous Step 3. Here, glass eels had the preference to orient 359°, towards the magnetic direction of the flow (mean direction indicated by the black arrow, with the dashed lines indicating the 95% confidence intervals).

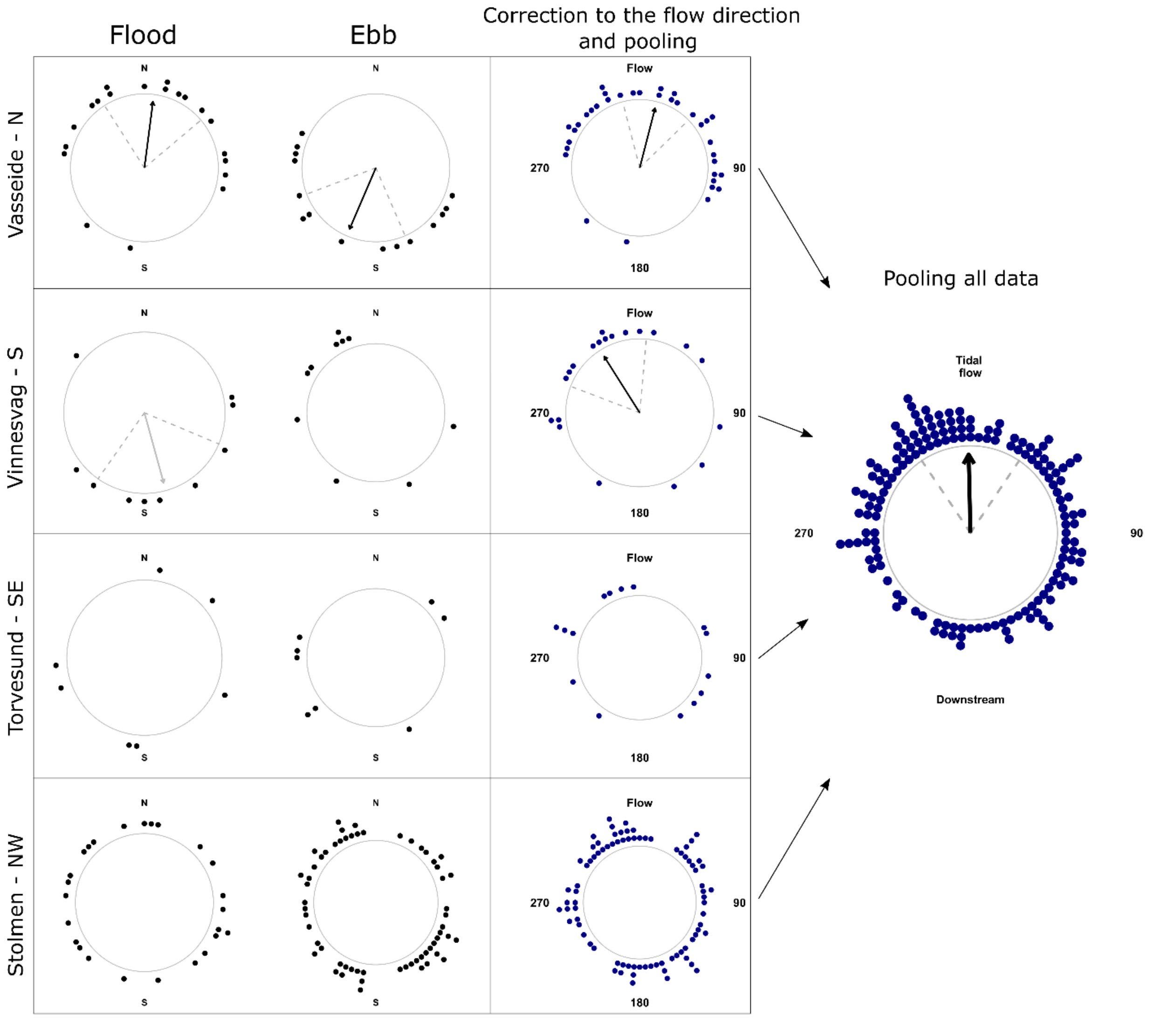

Supplementary Figure 4. Magnetic orientation with respect to the magnetic North and the magnetic direction of the tidal flow. The magnetic orientation of the glass eels (*Anguilla anguilla*) according to the collection site and the tide (ebb/flood) is shown. Black data points are the orientation directions with respect to the magnetic field in the lab (red North in Supplementary Figure 2). Navy blue data points show the magnetic orientation with respect to the magnetic direction of the flow (0° = upstream, 180° = downstream). The final plot is the same displayed in Fig. 3 in the main text, in which all the flow-corrected data are pooled together.

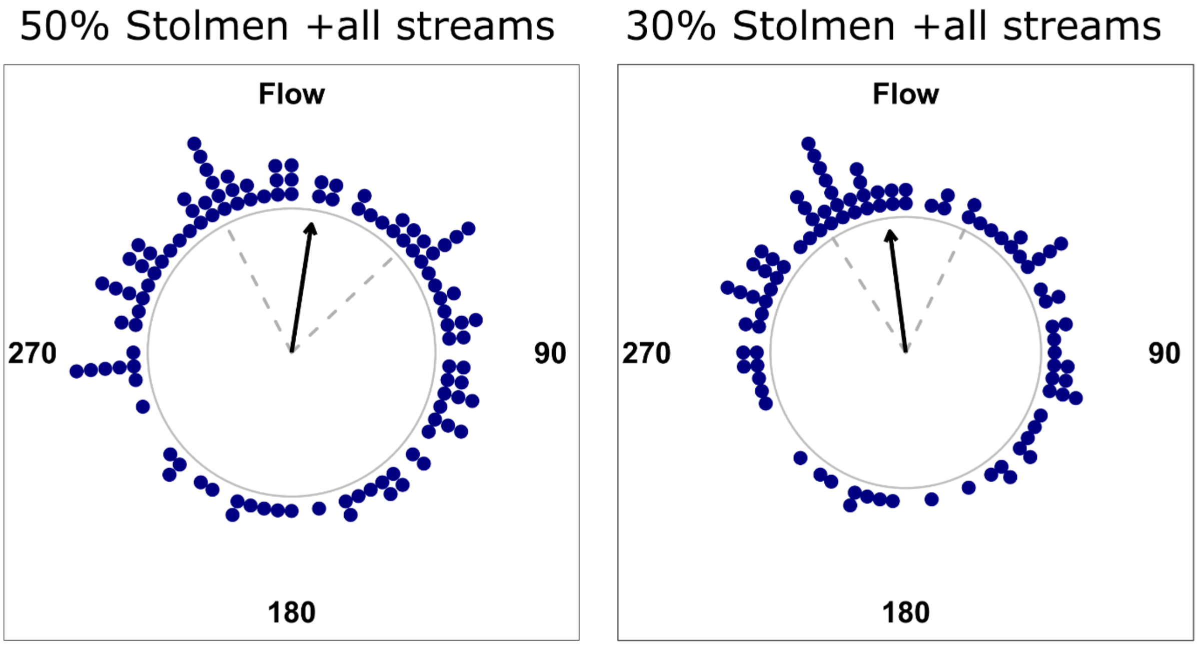

Supplementary Figure 5. Relative contribution of glass eels from the Stolmen estuary to the results. In the figure above, the data displayed in Figure 3 (main text) are shown, but with a random subsampling of data of the glass eels collected at Stolmen. This collection site had the highest abundance of glass eels, and this analysis show how the results vary considering only random 50% and 30% of the eels collected at Stolmen. The results do not change compared to those displayed in Figure 3, and glass eels orient against the magnetic direction of tidal currents. With 50% Stolmen: N= 112, mean = 9°, r = 0.2, p = 0.006; With 30% Stolmen: N= 92, mean = 352°, r = 0.3, p = 0.0005
