# Supplementary material for: Glass eels (Anguilla anguilla) imprint the magnetic direction of tidal currents from their juvenile estuaries
Source: Commun Biol. 2019 Oct 8;2:366. doi: 10.1038/s42003-019-0619-8 (PMC6783477; doi:10.1038/s42003-019-0619-8)
Supplement: Supplementary file 2 — Description of additional supplementary items [file 42003_2019_619_MOESM2_ESM.docx]

**Description of additional supplementary items**

**Supplementary data 1.** Date and time of the tests, location and direction of the stream where the eels (*Anguilla anguilla*) were collected, and the direction of the magnetic north in the laboratory during each test. Each row corresponds to a test of one individual glass eel. Details of the experiments with the 173 glass eels newly tested are included here while details of the tests of re-analyzed data are in the SI of Cresci et al. 2017 (<http://advances.sciencemag.org/content/3/6/e1602007>). The locations where eels were collected are: Vasseide (60.1122 N and 5.2298 E, flowing to the north), Torvesund (60.0294 N, 5.3016 E, flowing to the southeast), Vinnesvåg (60.0088 N, 5.2583 E, flowing to the south), and Stolmen (60.0082 N and 5.0788 E, flowing to the northwest).

**Supplementary data 2.** Mean bearing in decimal degree (°) and Rayleigh’s r value of each data point displayed in Figure 3. Each data point corresponds to the bearing of one glass eel.
