# Supplementary material for: Glass eels (Anguilla anguilla) imprint the magnetic direction of tidal currents from their juvenile estuaries
Source: Commun Biol. 2019 Oct 8;2:366. doi: 10.1038/s42003-019-0619-8 (PMC6783477; doi:10.1038/s42003-019-0619-8)
Supplement: Supplementary file 5 — Reporting Summary [file 42003_2019_619_MOESM5_ESM.pdf]

nature research

Corresponding author(s): Alessandro Cresci

Last updated by author(s): Sep 5, 2019

Reporting Summary

Nature Research wishes to improve the reproducibility of the work that we publish. This form provides structure for consistency and transparency in reporting. For further information on Nature Research policies, see Authors & Referees and the Editorial Policy Checklist.

Statistics

For all statistical analyses, confirm that the following items are present in the figure legend, table legend, main text, or Methods section.

n/a	Confirmed	
☐	☒	The exact sample size (*n*) for each experimental group/condition, given as a discrete number and unit of measurement
☐	☒	A statement on whether measurements were taken from distinct samples or whether the same sample was measured repeatedly
☐	☒	The statistical test(s) used AND whether they are one- or two-sided<br>*Only common tests should be described solely by name; describe more complex techniques in the Methods section.*
☒	☐	A description of all covariates tested
☐	☒	A description of any assumptions or corrections, such as tests of normality and adjustment for multiple comparisons
☐	☒	A full description of the statistical parameters including central tendency (e.g. means) or other basic estimates (e.g. regression coefficient) AND variation (e.g. standard deviation) or associated estimates of uncertainty (e.g. confidence intervals)
☐	☒	For null hypothesis testing, the test statistic (e.g. *F*, *t*, *r*) with confidence intervals, effect sizes, degrees of freedom and *P* value noted<br>*Give P values as exact values whenever suitable.*
☒	☐	For Bayesian analysis, information on the choice of priors and Markov chain Monte Carlo settings
☒	☐	For hierarchical and complex designs, identification of the appropriate level for tests and full reporting of outcomes
☒	☐	Estimates of effect sizes (e.g. Cohen's *d*, Pearson's *r*), indicating how they were calculated

Our web collection on statistics for biologists contains articles on many of the points above.

Software and code

Policy information about availability of computer code

Data collection	R version 3.5.1; RStudio Version 1.0.153
Data analysis	data collection and analysis was performed with the R package "discr" (https://github.com/jiho/discr)

For manuscripts utilizing custom algorithms or software that are central to the research but not yet described in published literature, software must be made available to editors/reviewers. We strongly encourage code deposition in a community repository (e.g. GitHub). See the Nature Research guidelines for submitting code & software for further information.

Data

Policy information about availability of data

All manuscripts must include a data availability statement. This statement should provide the following information, where applicable:

- Accession codes, unique identifiers, or web links for publicly available datasets
- A list of figures that have associated raw data
- A description of any restrictions on data availability

The single data points that support the findings of this study are all displayed in Figure 3 in the main manuscript and Supplementary table 2

Field-specific reporting

Please select the one below that is the best fit for your research. If you are not sure, read the appropriate sections before making your selection.

☒ Life sciences ☐ Behavioural & social sciences ☐ Ecological, evolutionary & environmental sciences

For a reference copy of the document with all sections, see nature.com/documents/nr-reporting-summary-flat.pdf

Life sciences study design

All studies must disclose on these points even when the disclosure is negative.

Sample size	As the study is based on the observation of the behavior of eels collected in the wild, the sample size was dependent on the quantity of animals that were successfully collected. The sample size was considered sufficiently large based on the sample size requirements of the statistical analysis used in the study (Rayleigh's test of uniformity). For this statistics a sample size >30 is preferred to detect patterns and in the present study we relied on a sample of 222 animals.
Data exclusions	No data were excluded
Replication	This study is a first observation of a magnetic orientation entrained by tidal currents, thus no replication of this experiment has been performed to date. However, the results reported in this study confirm the finding of a tidal-dependent magnetic orientation in glass eels reported in Cresci et al., 2017 (https://advances.sciencemag.org/content/3/6/e1602007)
Randomization	The experimental animals used in the study were categorized based on their recruitment estuary, which were oriented towards different cardinal directions in order to maximize randomization.
Blinding	Data on the orientation of the animals were collected blindly (i.e. without verifying the collection site of the animal at the time of the video tracking procedure).

Reporting for specific materials, systems and methods

We require information from authors about some types of materials, experimental systems and methods used in many studies. Here, indicate whether each material, system or method listed is relevant to your study. If you are not sure if a list item applies to your research, read the appropriate section before selecting a response.

Materials & experimental systems

n/a	Involved in the study	
☒	☐	Antibodies
☒	☐	Eukaryotic cell lines
☒	☐	Palaeontology
☐	☒	Animals and other organisms
☒	☐	Human research participants
☒	☐	Clinical data

Methods

n/a	Involved in the study	
☒	☐	ChIP-seq
☒	☐	Flow cytometry
☒	☐	MRI-based neuroimaging

Animals and other organisms

Policy information about studies involving animals; ARRIVE guidelines recommended for reporting animal research

Laboratory animals	The study did not involve laboratory animals
Wild animals	The animals used in the study were wild-caught glass eels of European eel (Anguilla anguilla). It was not possible to determine sex and age of the animals. The glass eels were collected in Austevoll, Norway, before their upstream freshwater migration between March and June of 2015, 2016 and 2017. They were caught using hand nets searching under rocks and sediment at low tide. No fish were harmed while performing the experiments, and after use, they were either returned to the wild or sacrificed humanely.
Field-collected samples	Glass eels were kept in 20 L maintenance tanks, where they were re-acclimated to near full salinity seawater (32 ppt) after capture. They were kept in aerated aquaria in a temperature-controlled room set to ambient conditions similar to those of the local Langenuen fjord (ranging between 6 and 10 °C). Animals were not fed (they were at the pre-feeding stage) and were kept in 14 h light and 10 h dark cycle (following the daylength at the study location during the observation period). Two-thirds of the volume of each aquarium was replaced with filtered seawater every 48 hours to maintain water quality. The seawater was provided by the filtration system at the Institute of Marine Research's Austevoll Research Station, which collects seawater from the Langenuen fjord at a depth of 160 m.
Ethics oversight	At the time that this study was conducted, no permits were required by the Norwegian authorities because no fish were harmed while performing the experiments, and after use, they were either returned to the wild or sacrificed humanely. Eels were collected under research catch permit #11/11448 issued by the Norwegian Fiskeridirektorat.

Note that full information on the approval of the study protocol must also be provided in the manuscript.
